# Influence of the Arrangement of Surgical Light Axes on the Air Environment in Operating Rooms

**DOI:** 10.1155/2019/4861273

**Published:** 2019-03-26

**Authors:** Tetsuya Kai, Nobuyasu Ayagaki, Hidekazu Setoguchi

**Affiliations:** ^1^Anesthesiology and Clinical Research Institute, National Hospital Organization Kyushu Medical Center, Fukuoka 810-8563, Japan; ^2^Central Uni Co., Ltd., Fukuoka 802-0823, Japan; ^3^Operating Rooms, Kyushu University Hospital, Fukuoka 812-8582, Japan

## Abstract

**Purpose:**

Surgical lights in the operating rooms are typically installed in a single axis in the center of the room or in two axes on both sides of the operating table. In the single-axis installation, the air-conditioning outlet cannot be placed in the center of the ceiling, which may affect the air current. Therefore, we measured the air current and cleanliness in two equivalent operating rooms using a vertical laminar airflow system equipped with either single-axis or double-axis surgical lights.

**Methods:**

Air current was measured using a three-dimensional ultrasonic anemometer. Cleanliness was evaluated by measuring the amount of dust before and after air-conditioner activation. To visualize the air current, smoke was illuminated on a sheet of laser light while the air-conditioning was stopped, and changes after air-conditioning activation were observed.

**Results:**

In the single-axis room, an oblique fast air current flowing from the surrounding air outlet toward the center was observed, and the flow velocity fluctuated greatly. In the double-axis room, uniform downward laminar airflow was observed. The amount of dust at the center decreased significantly faster in the double-axis room; thus, the cleanliness at the center was higher in the double-axis room. Persistent stagnation of smoke was observed below the single-axis lighting, whereas smoke below the double-axis lighting was immediately dispersed and the air cleared even when surgical lights were in the position for surgery.

**Conclusion:**

Uniform vertical laminar airflow was formed and high cleanliness was achieved in the center of the room when the surgical lights were arranged in two axes.

## 1. Introduction

Once surgical site infection occurs, it becomes a great burden in the postoperative course, lengthens the hospital stay, and increases the cost of postoperative care [1]. Surgical site contamination is chiefly attributed to airborne particles, which may carry microorganisms [2]. Therefore, air quality in the operating room is important to prevent surgical site infection.

In order to provide the clean air environment at the surgical field, the ideal ventilation system for operating rooms was thought to be a vertical laminar airflow system that supplies clean air from an outlet on the ceiling and collects air from exhaust grilles located on the wall [1–4]. Although the infection protective effect of the laminar airflow has been questioned and disputed recently [5, 6], there are still compiling evidence that shows the laminar airflow decreases microbial contamination in the surgical field [7, 8].

It has been revealed that the air current in the operating room with a vertical laminar airflow system is influenced, leading to the contamination of surgical field, by many factors including surgical light [9–11], surgical staffs [9], and door opening [12]. When we investigated the influence of the surgical light on the vertical laminar airflow, we happened to notice that the stagnation of air current occurred directly below the axis of surgical light.

Surgical lights are typically installed in a single axis in the center of the room or in two axes arranged on both sides of the operating table. In the single-axis installation, since the surgical light axis is at the center of the ceiling, the air-conditioning outlet must be arranged around the axis. Therefore, the air outlet is not installed directly above the center of the operating table, which is most frequent position of the surgical field. Thus, the air current above the operating table may vary dramatically between single-axis and double-axis surgical light installations. However, no report examining the differences between these conditions could be found in our search.

This study aimed at examining the influence of the arrangement of surgical light axes on the air environment around the operating table by analyzing air currents and assessing cleanliness near the operating table in two equivalent operating rooms using a vertical laminar airflow system equipped with either single- or double-axis surgical lights.

## 2. Methods

### 2.1. Location

We took measurements in two operating rooms of a university hospital with the same room shape and air-conditioning outlet shape, as well as nearly equivalent air-conditioning design. Operating Room 17 had a single-axis surgical light installation, and Operating Room 16 had a double-axis installation. While the basic type of surgical lighting in this hospital uses a double-axis installation, Room 17 has a ceiling-suspended microscope; thus, surgical lights are installed in a single axis to avoid interference of arms. These rooms both have Cleanliness Class 1,000 air-conditioning design (less than 1,000 airborne particles of diameter 0.5 *µ*m or larger per cubic foot; ISO Class 6), and the shape and volume of the rooms are identical, with the same outer shape of the air-conditioning outlet. However, Room 17 with single-axis lights at the center is designed to have a slightly larger airflow volume in consideration of heat generation from devices used in the room, including the microscope, and thus has a slightly higher flow velocity at the outlet (Table 1).

For measurements, surgical lights and other ceiling-mounted arms were moved away from the laminar airflow area; however, the surgical lights were placed either away from the laminar airflow area or in the appropriate position for surgery in the air current visualization study. Air-conditioning was operated at a temperature setting of 25°C.

### 2.2. Three-Dimensional Airflow Velocity Measurement

**Figure 1:**
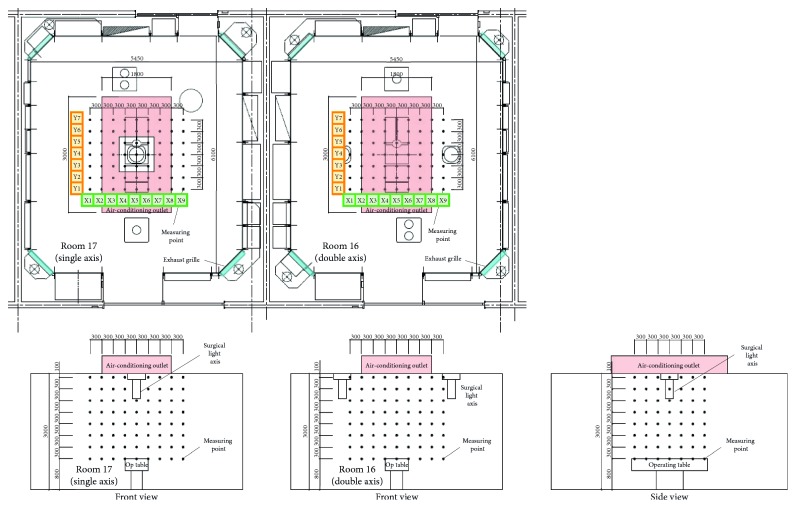
Three-dimensional airflow velocity measurement points. Airflow was measured at 504 points around the operating table at 300-mm intervals (except for points that interfered with the surgical light axes and arms): 9 points in the right and left directions (*X* axis) × 7 points in the front and rear directions (*Y* axis) × 8 points in the vertical direction.

We measured the three-dimensional airflow direction and velocity using an ultrasonic clean room anemometer (model WA-790; Sonic Corporation, Tokyo, Japan). With the operating table at the center, measurements were taken at 504 points, with 9 in the right and left directions (*X* axis) × 7 in the front and rear directions (*Y* axis) × 8 in the vertical direction, at a distance of 300 mm from each other. The points that overlapped with surgical light axes and arms were omitted as it was impossible to take measurements at these locations (Figure 1).

### 2.3. Cleanliness Assessment

**Figure 2:**
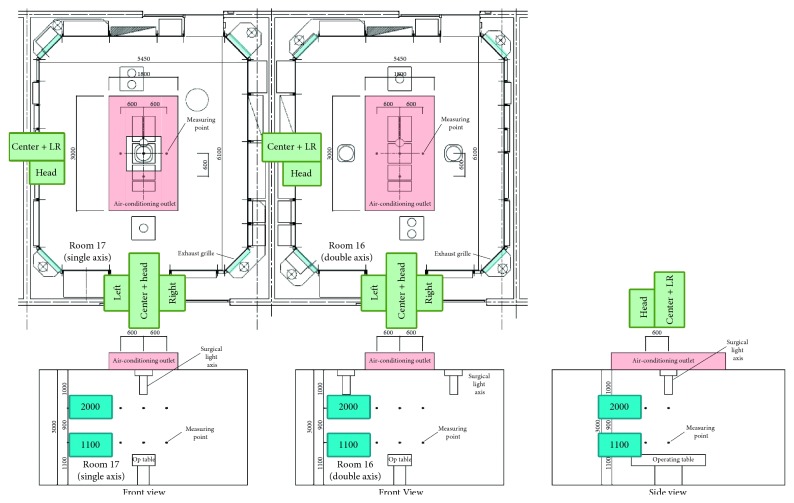
Cleanliness assessment points. Cleanliness was assessed at 8 points: center, head, right, and left at heights of 1,100 and 2,000 mm.

Cleanliness was evaluated with a cleanliness recovery test, in which dust was artificially generated with the air-conditioner stopped, and the dust removal process was assessed after air-conditioner activation. Using a particle counter (MetOne 3413; Hach Company, Loveland, CO, USA), suction sampling was performed at 1 cubic foot/min to measure the quantity of dust particles with a grain size of 0.5 *μ*m or larger. Air-conditioner operation was started with an initial dust particle load of 70,000 to 80,000/cubic foot, and transitions in the quantity of dust particles were measured at 1 min intervals thereafter for 10 min. Measurements were made at the center of the operating table, the head, and on the right and left sides, with 4 each at heights of 1,100 mm and 2,000 mm, for a total of 8 points (Figure 2). The heights of 1,100 mm and 2,000 mm correspond to the general operating filed height and the overhead height, respectively.

### 2.4. Air Current Visualization

To observe the air current, particles were illuminated using smoke and laser lights. A harmless smoke of fine particles (with a diameter of around 10 *µ*m) was created using a glycol-based solvent and water with a portable vapor generator (Porta Smoke PS-2005; Dainichi Co., Ltd., Niigata, Japan). This smoke was illuminated by two laser light sheet sources (Parallel Eye H; Shin Nippon Air Technologies Co., Ltd., Tokyo, Japan) placed facing each other. Smoke was created on a sheet of laser light while the air-conditioning was stopped, and changes in the smoke after air-conditioning activation were observed and recorded. The study was performed with surgical lights placed either away or in the appropriate position for surgery.

### 2.5. Statistical Analysis

To compare the cleanliness, the cleanliness recovery processes of each corresponding measurement point of both rooms were analyzed with the Wilcoxon matched-pairs signed-rank test, using IBM SPSS Statistics version 22. Statistical significance was defined as a *P* value <0.05.

## 3. Results

### 3.1. Three-Dimensional Airflow Velocity Measurement

**Figure 3:**
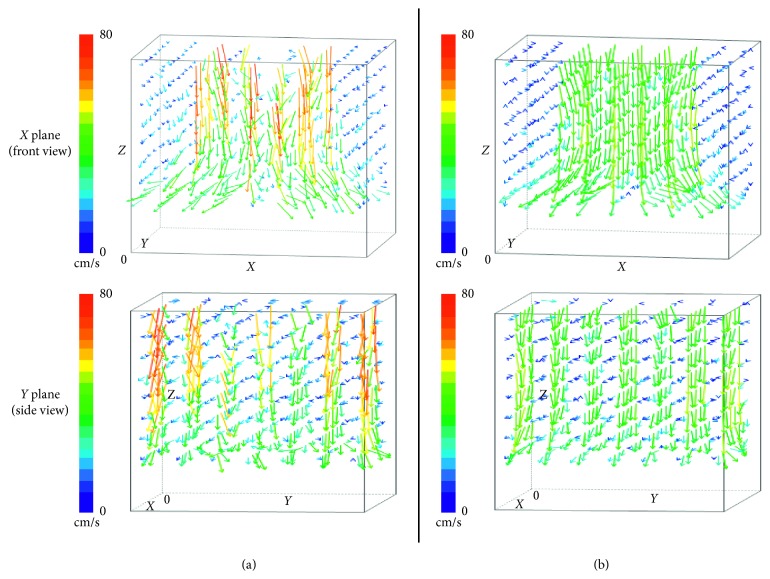
Three-dimensional perspective views of the air current. X plane (front view): 3D perspective views looking at the operating table from the head. Y plane (side view): 3D perspective views looking at the operating table from the right side. The arrowheads indicate airflow direction, and the color and length of the arrows indicate airflow velocity. (a) Single-axis room. (b) Double-axis room.

Airflow direction and velocity are shown in three-dimensional perspective views (Figure 3). The upper panels are front views looking at the operating table from the head, and the lower panels are side views looking at the operating table from the right side. In the single-axis room, as the air outlet was located away from the center and the effective outlet area was small, the velocity at the vicinity outlet was high, and an oblique air current flowing toward the center was observed. Moreover, there were large fluctuations in flow velocity in the single-axis room. On the contrary, in the double-axis room, the air current showed downward laminar flow with uniform velocity.

When we looked at the airflow direction and velocity in sagittal plane (Supplementary 1, Figure 1S) and coronal plane (Supplementary 1, Figure 2S) of the operating table, we found that single-axis installation caused greater fluctuations in airflow velocity, with an oblique air current flowing toward the center of the room from its proximity.

**Figure 4:**
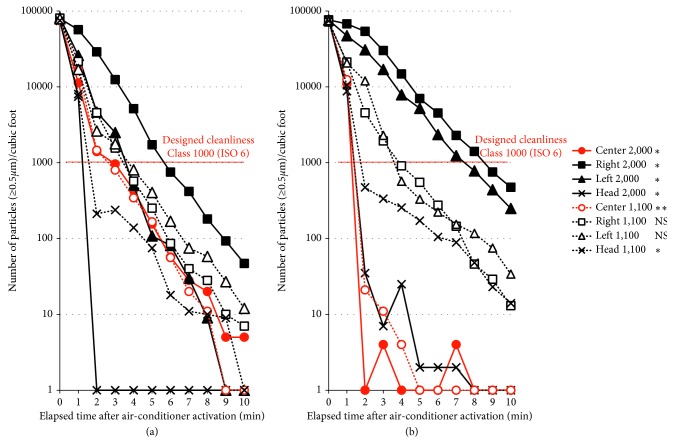
Cleanliness assessment results. The reduction process of the number of artificially generated dust particles (≥0.5 *µ*m) per cubic foot after air-conditioner activation measured every minute at 8 points are shown. In the comparison between the rooms at corresponding measurement points, ^*∗*^ and ^*∗∗*^ indicate significant difference (^*∗*^, *P* < 0.05; ^*∗∗*^, *P* < 0.01). (a) Single-axis room. (b) Double-axis room.

**Figure 5:**
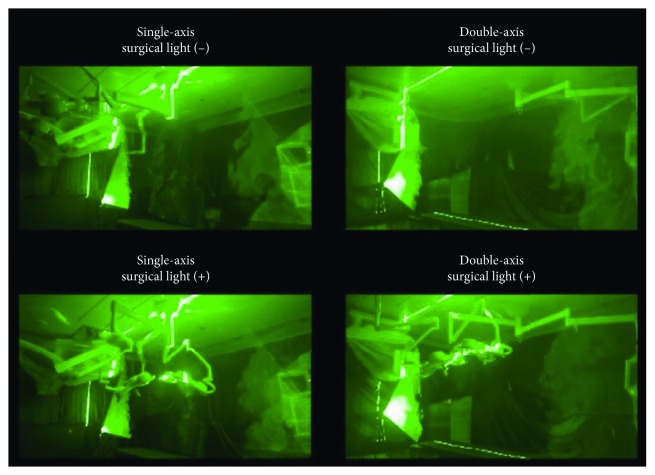
Air current visualization. Air current was visualized using smoke and laser light in the single-axis and double-axis room with surgical lights placed either away (−) or in the appropriate position for surgery (+).

When airflow direction and velocity were observed on a horizontal plane at a height of 2,900 mm (Supplementary 1, Figure 3S), there was no airflow directly under the center of the ceiling, and the airflow velocity in its proximity was faster in the single-axis room, as there was no outlet at the center of the ceiling. In contrast, the airflow velocity was nearly uniform in the double-axis room. Although there were larger fluctuations in airflow velocity in the single-axis room on a horizontal plane at a height of 2,000 mm (Supplementary 1, Figure 4S), the difference in airflow velocity was not obvious at a height of 1,100 mm, which corresponds to the height of the general surgical field (Supplementary 1, Figure 5S).

The average air current temperature at the outlet during airflow velocity measurement was 23.4°C in the single-axis room and 23.1°C in the double-axis room.

### 3.2. Cleanliness Assessment

The results of the cleanliness recovery test at 8 points in each room are plotted on a graph (Figure 4). Although the number of particles per cubic foot decreased to less than 1,000 within 4 min in both rooms at a height of 1,100 mm, which corresponds to the surgical field, 6 min were required in the single-axis room and 9 min in the double-axis room at a height of 2,000 mm to achieve less than 1,000 per cubic foot. At the center of the room (shown as red lines in Figure 4), the number of particles per cubic foot dropped below 1,000 in 3 min and below 100 in 6 min in the single-axis room, whereas the number of particles dropped below 100 in 2 min and below 10 in 4 min in the double-axis room, which was significantly different between the rooms both at the heights of 1,100 and 2,000 mm, indicating that the cleanliness at the center was clearly greater in the double-axis room.

### 3.3. Air Current Visualization

In the single-axis room, persistent stagnation of smoke was observed below the axis, indicating that the air current at the center of the room below the axis was stagnant (Figure 5 and Supplementary 2). In the double-axis room, smoke was immediately dispersed by the uniform downward air current, and the air cleared even when the surgical lights were in the position for surgery.

## 4. Discussion

To examine the effects of surgical light installation conditions on the air environment in operating rooms, we implemented three-dimensional airflow velocity measurement and a cleanliness assessment in two operating rooms using single-axis or double-axis installations; room size and structure were identical, and the air-conditioning designs were similar. Since the two rooms compared in this study were designed with an identical air outlet outer size, the single-axis room without the outlet at the center had a smaller effective outlet area. The combination of this factor and a larger airflow volume design made the outlet airflow velocity in the single-axis room much greater than that in the double-axis room. Therefore, in the single-axis room, the rapid airflow occurred flowing toward the center of the room from its proximity in an oblique direction, as the distance from the ceiling was greater, resulting in sufficient downward airflow at the height of the surgical field. Meanwhile, precise downward vertical laminar airflow with nearly uniform velocity was formed in the double-axis room, and cleanliness at the center of the room was significantly greater in the double-axis room, even though the airflow volume was designed to be smaller than in the single-axis room.

In the present study, we used the particle amount as a surrogate indicator of cleanliness since microorganisms are carried by airborne particles and it has been reported that the number of particles correlates to microbial counts [13].

A limitation of this study was that actual operating conditions were not simulated so that surgical team members, a patient on the table, surgical drapes, and instrument tables were not in existence, and surgical light bodies were placed away from the operating table. Air current is influenced by these factors. One of the reasons why we did not simulate the clinical conditions was that the aim of this study was to simply clarify how the centrally located surgical light axis influences the air currents in the operating room with the laminar airflow system. Another reason was that, for three-dimensional airflow velocity measurement, it is practically nearly impossible to set the spatial measurement points when obstacles such as medical staffs and surgical light bodies exist. Even with such limitation, this study found that vertical laminar airflow was clearly different between single-axis and double-axis surgical light installations and the cleanliness at the center was significantly high in the double-axis installation. Moreover, the air current visualization study confirmed that the dust in the surgical field was eventually cleared in the double-axis room even when surgical light bodies were placed above the surgical field mimicking actual operating condition, whereas the dust persisted for long above the operating table under the light axis in the single-axis room.

Although the cleanliness recovery in the center was significantly faster in the double-axis room, it tended to be slower at points on the right and left at a height of 2 m in the double-axis room. Since these points are at the fringe of laminar airflow and the velocities are slower compared to the single-axis room, they are assumed to be prone to the influence of dust outside the airflow. Recovery of cleanliness at the height of the surgical field was faster in both the right and left positions, which was not different between the rooms. This was probably because the laminar airflow spreads slightly at this height. Because of the fact that the air current in the area outside the laminar airflow is weak, as proven in this study, and the cleanliness in the area outside the laminar airflow is reported to be poor [14], it is important to place the surgical instrument table within the laminar airflow and maintain the cleanliness of the sterile instruments to prevent contamination during surgery in operating rooms with the laminar airflow system.

Laminar airflow systems were claimed to be effective in prevention of postoperative infection by reducing airborne particles in the proximity of the surgical field in procedures requiring a high degree of cleanliness, such as joint replacement surgery [15]. However, some recent reports claimed that laminar airflow systems were associated with more cases of surgical site infection as compared with conventional ventilation systems [5, 6]. The World Health Organization 2016 recommendations for prevention of surgical site infection stated that laminar airflow systems should not be used as a preventive measure to reduce the risk of infection in joint replacement surgery [16]. The reason given was that laminar airflow systems have relatively high installation and operation costs, with no clear evidence of benefit, compared with conventional ventilation systems. Although the recommendation does not suggest the use of laminar airflow systems for prevention of infection, it does not recommend against their use [17].

Laminar airflow has been considered a risk for temperature reduction in the surgical site tissue and resulting whole-body hypothermia, as the cool air current directly strikes the surgical site [18]. Hypothermia reduces immunocompetence and increases the risk of postoperative infection [16]. It is possible that this mechanism led to the finding of a meta-analysis stating that surgical site infection increases under laminar airflow [6]. However, as there are no data on body temperature during surgery in a report that claimed laminar airflow increased the risk of surgical site infection [5], the effect on body temperature is unclear. The risk of postoperative infection due to hypothermia is a consequence of failure to maintain body temperature. Hypothermia can be prevented if active heating is implemented, even in an operating room with laminar airflow, as we usually do with forced air warming. We therefore do not consider laminar airflow as a risk for infection. In addition, it has recently been proven that a forced air warming does not disturb airflow around surgical field under the laminar airflow system [19]. A study that investigated the number of airborne bacteria during orthopedic surgery in operating rooms with or without laminar airflow reported that bacterial counts were significantly higher in operating rooms without laminar airflow and that the type of warming device (forced air or nonforced air) did not affect the bacterial counts [7]. This finding suggests that laminar airflow itself does not increase the risk of infection but instead prevents infection. A recent report that compared newly developed temperature-controlled airflow ventilation with laminar airflow and turbulent mixed airflow showed that, in terms of the air cleanliness (as measured by bacterial colony-forming unit), laminar airflow was much higher than turbulent mixed airflow and temperature-controlled airflow was practically comparable with laminar airflow [20]. Since temperature-controlled airflow provides sufficient cleanliness with slower airflow, which may reduce the cooling effect and thus decrease the risk of patient hypothermia, it is suggested that temperature-controlled airflow could be an alternative ventilation for operating rooms used for infection-sensitive surgery [20].

Our air current visualization study confirmed that the dust in the surgical field directly under the light axis in the single-axis room persisted for long, whereas the dust in the double-axis room disappeared shortly, even when surgical light bodies were placed above the surgical field. It has also been shown that air currents are greatly affected by the surgical light bodies in rooms with laminar airflow [10, 11]. It is possible that research results regarding the relation of laminar airflow and the risk of surgical site infection are inconsistent because of the effects of the surgical light axis, as shown in this study, as well as those of the lighting bodies. These factors should be considered in future research to determine the association between laminar airflow and surgical site infection.

Adopting conventional ventilation instead of laminar airflow, which is costly, has recently been recommended for new operating rooms [6, 21]. The concept of conventional ventilation is to reduce the overall number of particles by generating turbulence and homogenizing the air inside the operating room. To achieve this concept, air currents need to circulate throughout the entire room to homogenize the air, while the existence of airflow stagnation will impede its achievement. Whether laminar airflow or conventional ventilation is used, allowing the presence of numerous particles in air around the surgical field through stagnation of air current probably leads to surgical site infection and should be avoided since it was established that air microbial contamination is positively associated with the number of air particles [13]. Recently harmfulness of surgical smoke elicited by electrocautery to the surgical staff has become a problem [22]. Since air stagnation occurs at the center of the operating room under the surgical light axis in the single-axis room, it is highly possible for the surgical staff to inhale the more surgical smoke generated from the surgical field than in the double-axis room. Taking these factors in consideration, single-axis surgical light installation, which cannot have an air outlet at the center, should be avoided in vertical laminar airflow systems with ceiling outlets.

## 5. Conclusions

In operating rooms with a vertical laminar airflow system, uniform airflow was formed and higher cleanliness was achieved at the center of the room when the surgical lights were arranged in two axes.

## Figures and Tables

**Table 1:** Outlines of operating rooms.

	Single axis (No. 17)	Double axis (No. 16)
Width (m)	6.1	6.1
Distance (m)	5.45	5.45
Floor space (m^2^)	34.22	34.22
Ceiling height (m)	3.0	3.0
Room volume (m^3^)	102.7	102.7
Designed outdoor air intake volume (m^3^/h)	920	920
Designed total airflow volume (m^3^/h)	6,300	6,000
Air-conditioning outlet filter area (m^2^)	3.163	3.535
Designed outlet flow velocity (m/s)	0.553	0.471
Measured outlet flow velocity (m/s)	0.55	0.40

## Data Availability

The data used to support the findings of this study are included within the article and Supplementary Materials.

## Supplementary Materials

Supplementary 1 Figure 1S: air current direction and velocity in the central sagittal plane from the right side. The air current in the X-5 cross-sectional plane is shown by the direction and length of the arrows. Velocities are shown in cm/s as well as by the length and color of the arrows. The size of the red circles at measurement points indicates deviations in velocity. Red squares represent cleanliness assessment points. Figure 2S: air current direction and velocity in the central coronal plane from the head. The air current in the Y-4 cross-sectional plane is shown by the direction and length of the arrows. Velocities are shown in cm/s as well as by the length and color of the arrows. The size of the red circles at measurement points indicates deviations in velocity. Red squares represent cleanliness assessment points. Figure 3S: air current direction and velocity at a height of 2,900 mm. The air current in the horizontal plane at a height of 2,900 mm is shown by the direction and length of the arrows. Velocities are shown in cm/s, by the color of the panels, as well as by the length and color of the arrows. The size of the red circles at measurement points indicates deviations in velocity. Figure 4S: air current direction and velocity at a height of 2,000 mm. The air current in the horizontal plane at a height of 2,000 mm is shown by the direction and length of the arrows. Velocities are shown in cm/s, by the color of the panels, as well as by the length and color of the arrows. The size of the red circles at measurement points indicates deviations in velocity. Red squares represent cleanliness assessment points. Figure 5S: air current direction and velocity at a height of 1,100 mm. The air current in the horizontal plane at a height of 1,100 mm is shown by the direction and length of the arrows. Velocities are shown in cm/s, by the color of the panels, as well as by the length and color of the arrows. The size of the red circles at measurement points indicates deviations in velocity. Red squares represent cleanliness assessment points.

Supplementary 2 Video Clip: a video clip of the air current visualization study in the single-axis or double-axis room with surgical lights placed either away (−) or in the appropriate position for surgery (+). The video is fast-forwarded halfway.

Supplementary 3 Picture 1S: velocity measurement in double-axis room. Picture 2S: cleanliness assessment in single-axis room. Picture 3S: preparation for air current visualization in single-axis room. Picture 4S: preparation for air current visualization in double-axis room with lights in position.
