# Supplementary material for: Influence of the Arrangement of Surgical Light Axes on the Air Environment in Operating Rooms
Source: J Healthc Eng. 2019 Mar 26;2019:4861273. doi: 10.1155/2019/4861273 (PMC6458874; doi:10.1155/2019/4861273)
Supplement: Supplementary 1 — Figure 1S: air current direction and velocity in the central sagittal plane from the right side. The air current in the X-5 cross-sectional plane is shown by the direction and length of the arrows. Velocities are shown in cm/s as well as by the length and color of the arrows. The size of the red circles at measurement points indicates deviations in velocity. Red squares represent cleanliness assessment points. Figure 2S: air current direction and velocity in the central coronal plane from the head. The air current in the Y-4 cross-sectional plane is shown by the direction and length of the arrows. Velocities are shown in cm/s as well as by the length and color of the arrows. The size of the red circles at measurement points indicates deviations in velocity. Red squares represent cleanliness assessment points. Figure 3S: air current direction and velocity at a height of 2,900 mm. The air current in the horizontal plane at a height of 2,900 mm is shown by the direction and length of the arrows. Velocities are shown in cm/s, by the color of the panels, as well as by the length and color of the arrows. The size of the red circles at measurement points indicates deviations in velocity. Figure 4S: air current direction and velocity at a height of 2,000 mm. The air current in the horizontal plane at a height of 2,000 mm is shown by the direction and length of the arrows. Velocities are shown in cm/s, by the color of the panels, as well as by the length and color of the arrows. The size of the red circles at measurement points indicates deviations in velocity. Red squares represent cleanliness assessment points. Figure 5S: air current direction and velocity at a height of 1,100 mm. The air current in the horizontal plane at a height of 1,100 mm is shown by the direction and length of the arrows. Velocities are shown in cm/s, by the color of the panels, as well as by the length and color of the arrows. The size of the red circles at measurement points indicates deviations in [file 4861273.f1.pdf]

Figure 1s. Air current direction and velocity in the central sagittal plane

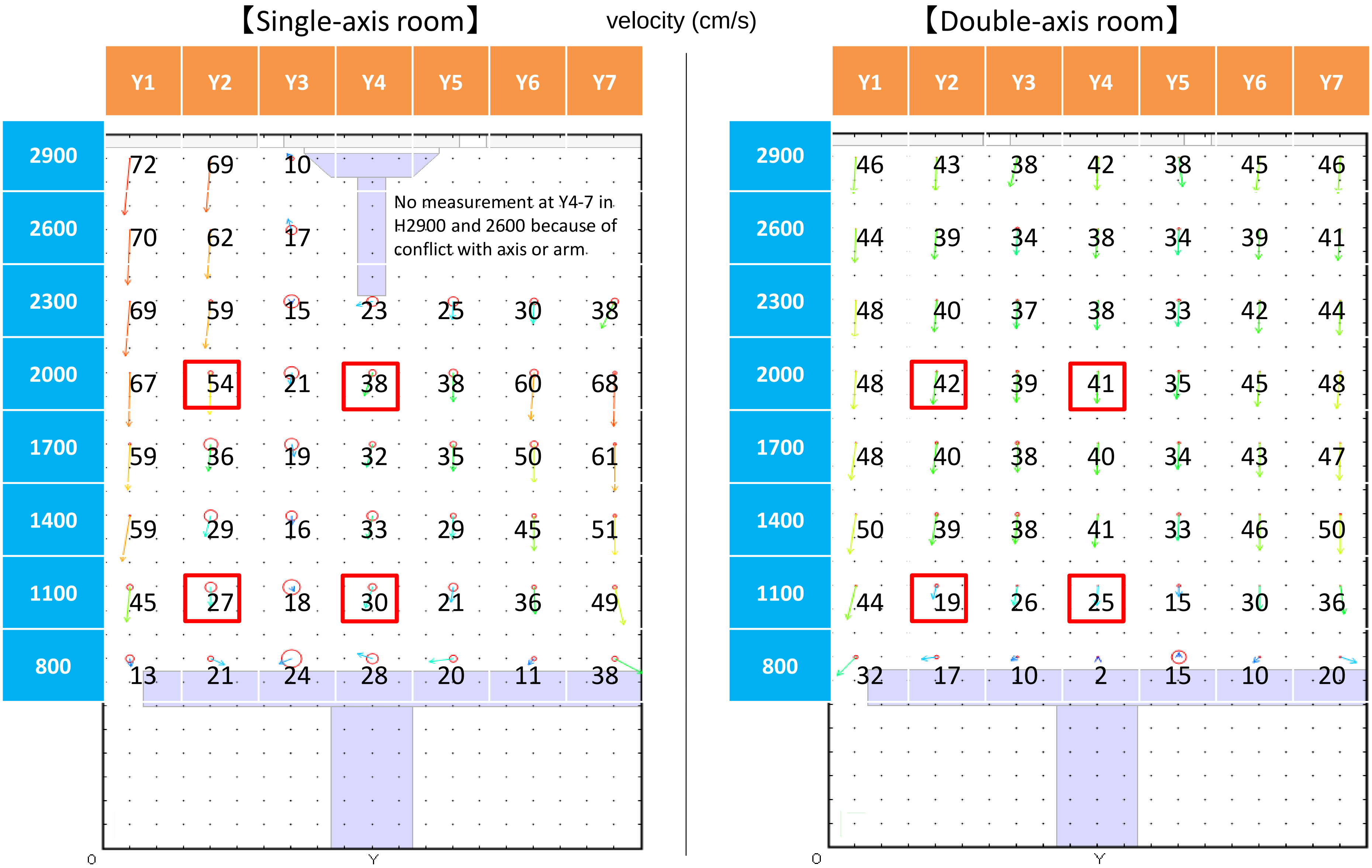

Figure 2s. Air current direction and velocity in the central coronal plane

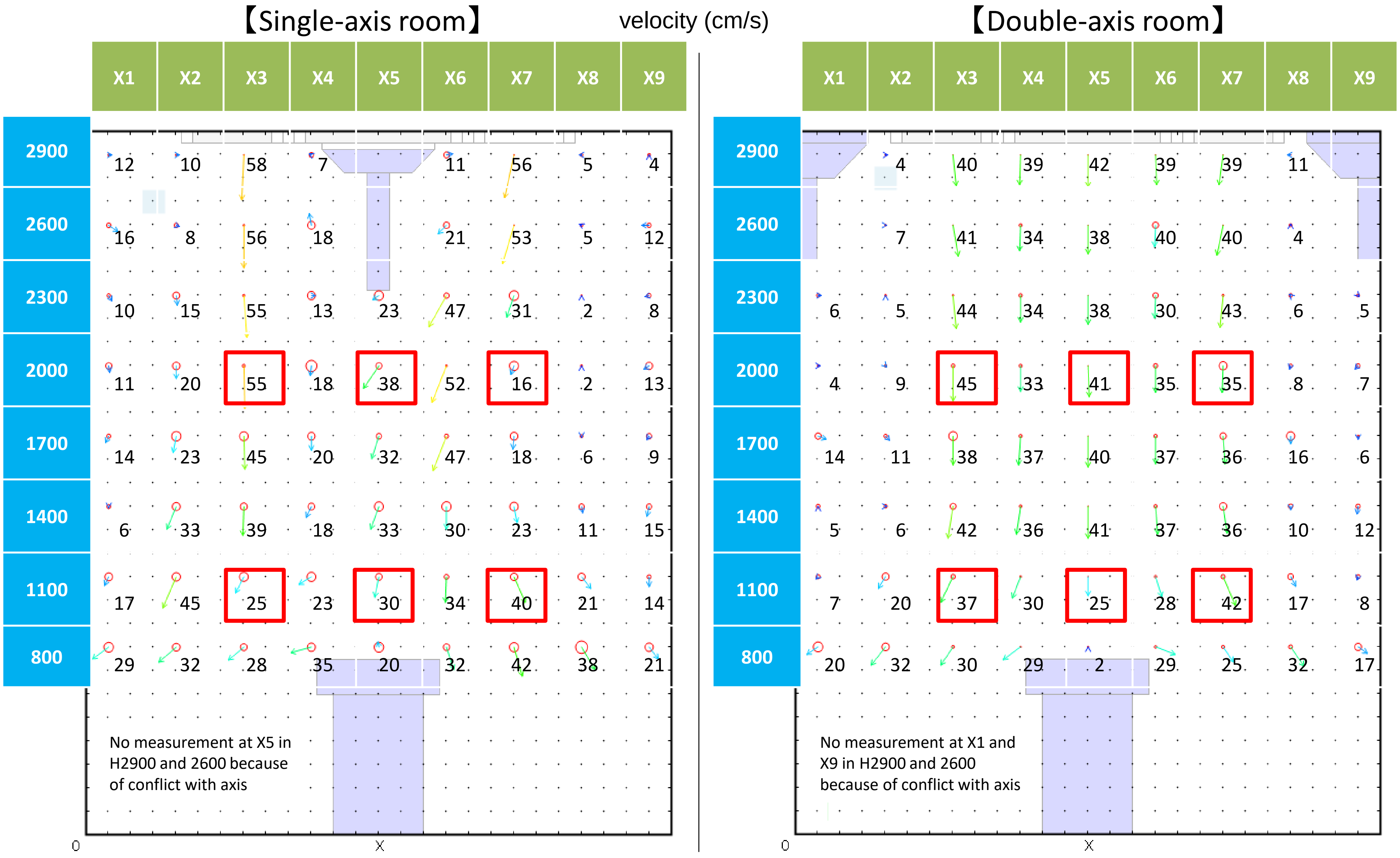

Figure 3s. Air current direction and velocity at a height of 2,900 mm

velocity (cm/s)

【Single-axis room】

	X1	X2	X3	X4	X5	X6	X7	X8	X9
Y7	12	11	69	55		60	64	7	5
Y6	11	9	68	50		59	60	10	7
Y5	11	12	46	6		13	43	9	9
Y4	12	10	58	7	Axis	11	56	5	4
Y3	4	5	26	8	10	12	23	8	5
Y2	5	6	60	47	69	49	62	8	5
Y1	9	11	67	32	72	57	62	7	5

【Double-axis room】

	X1	X2	X3	X4	X5	X6	X7	X8	X9
Y7	4	3	42	40	46	44	42	4	3
Y6	5	2	40	40	45	42	39	4	7
Y5	4	8	38	42	38	37	35	7	4
Y4	Axis	4	40	39	42	39	39	11	Axis
Y3	4	6	37	42	38	38	36	7	8
Y2	3	6	40	41	43	40	40	14	12
Y1	22	4	42	41	46	38	43	10	11

Figure 4s. Air current direction and velocity at a height of 2,000 mm

velocity (cm/s)

【Single-axis room】

	X1	X2	X3	X4	X5	X6	X7	X8	X9
Y7	12	10	62	46	68	57	64	11	5
Y6	12	5	48	46	60	62	56	11	14
Y5	11	14	25	30	38	37	18	8	12
Y4	11	20	55	18	38	52	16	2	13
Y3	15	15	48	15	21	38	55	23	15
Y2	15	15	55	52	54	52	58	30	18
Y1	12	16	64	42	67	65	58	26	15

0 X

【Double-axis room】

	X1	X2	X3	X4	X5	X6	X7	X8	X9
Y7	5	15	47	43	48	49	48	5	6
Y6	6	9	44	36	45	40	48	7	10
Y5	3	7	33	31	35	35	30	8	7
Y4	4	9	45	33	41	35	35	8	7
Y3	6	5	21	29	39	36	20	6	7
Y2	5	6	20	35	42	39	43	8	10
Y1	4	3	38	44	48	41	50	5	6

0 X

Figure 5s. Air current direction and velocity at a height of 1,100 mm

velocity (cm/s)

【Single-axis room】

	X1	X2	X3	X4	X5	X6	X7	X8	X9
Y7	10	13	41	42	49	54	47	21	7
Y6	8	8	32	26	36	44	45	24	7
Y5	13	31	30	28	21	33	40	20	14
Y4	17	45	25	23	30	34	40	21	14
Y3	15	45	27	29	18	28	33	41	12
Y2	14	24	34	29	27	26	35	21	14
Y1	10	24	32	29	45	48	34	19	10

【Double-axis room】

	X1	X2	X3	X4	X5	X6	X7	X8	X9
Y7	20	32	42	42	36	34	48	8	6
Y6	10	28	36	32	30	30	42	18	8
Y5	9	29	37	25	15	24	40	17	9
Y4	7	20	37	30	25	28	42	17	8
Y3	15	23	37	32	26	29	37	20	5
Y2	9	19	35	27	19	29	46	17	11
Y1	10	13	43	40	44	43	50	18	9
