# Supplementary material for: Influence of the Arrangement of Surgical Light Axes on the Air Environment in Operating Rooms
Source: J Healthc Eng. 2019 Mar 26;2019:4861273. doi: 10.1155/2019/4861273 (PMC6458874; doi:10.1155/2019/4861273)

Picture 1s. Velocity measurement in double-axis room

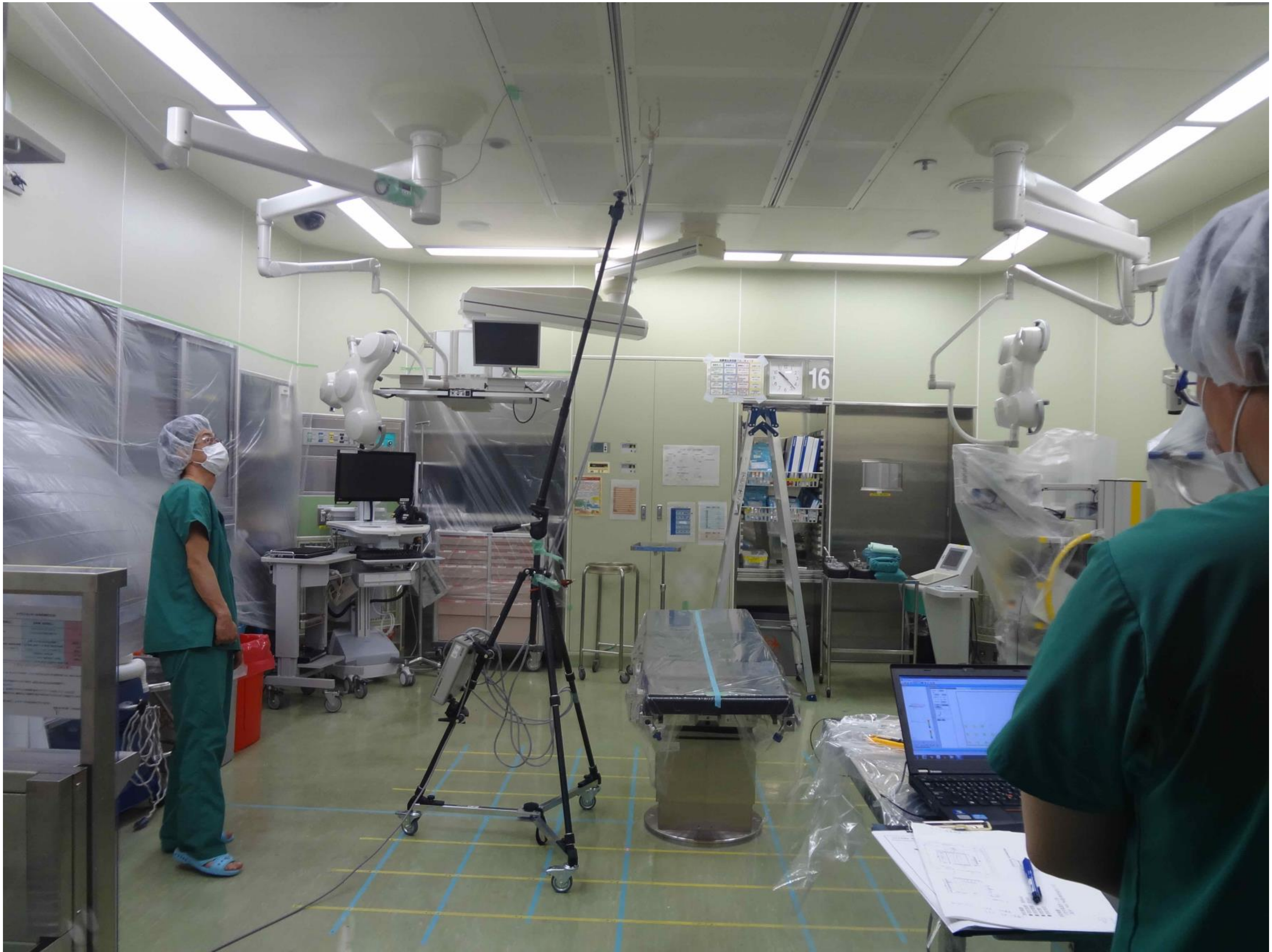

Picture 2s. Cleanliness assessment in single-axis room

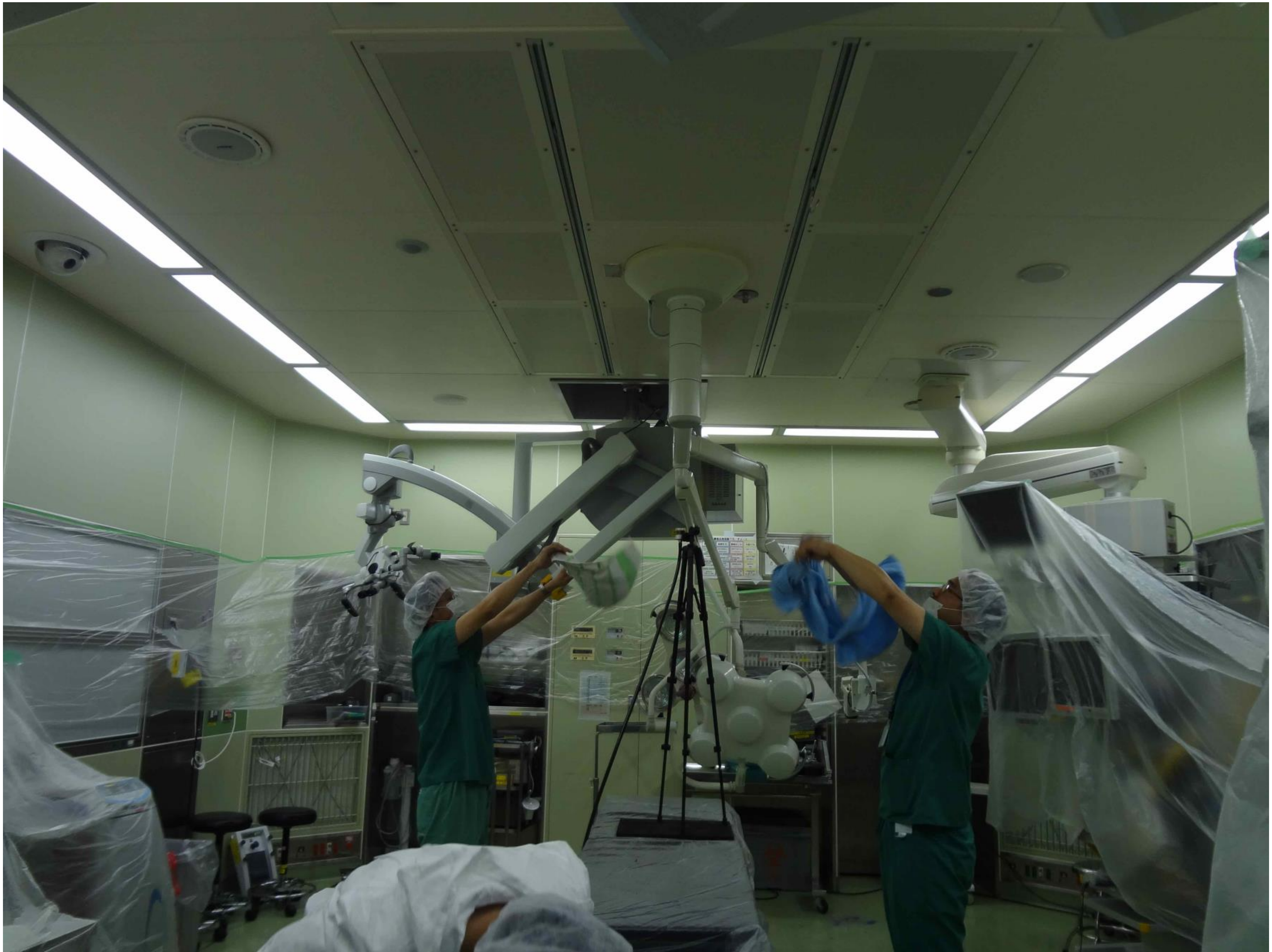

Picture 3s. Preparation for air current visualization in single-axis room

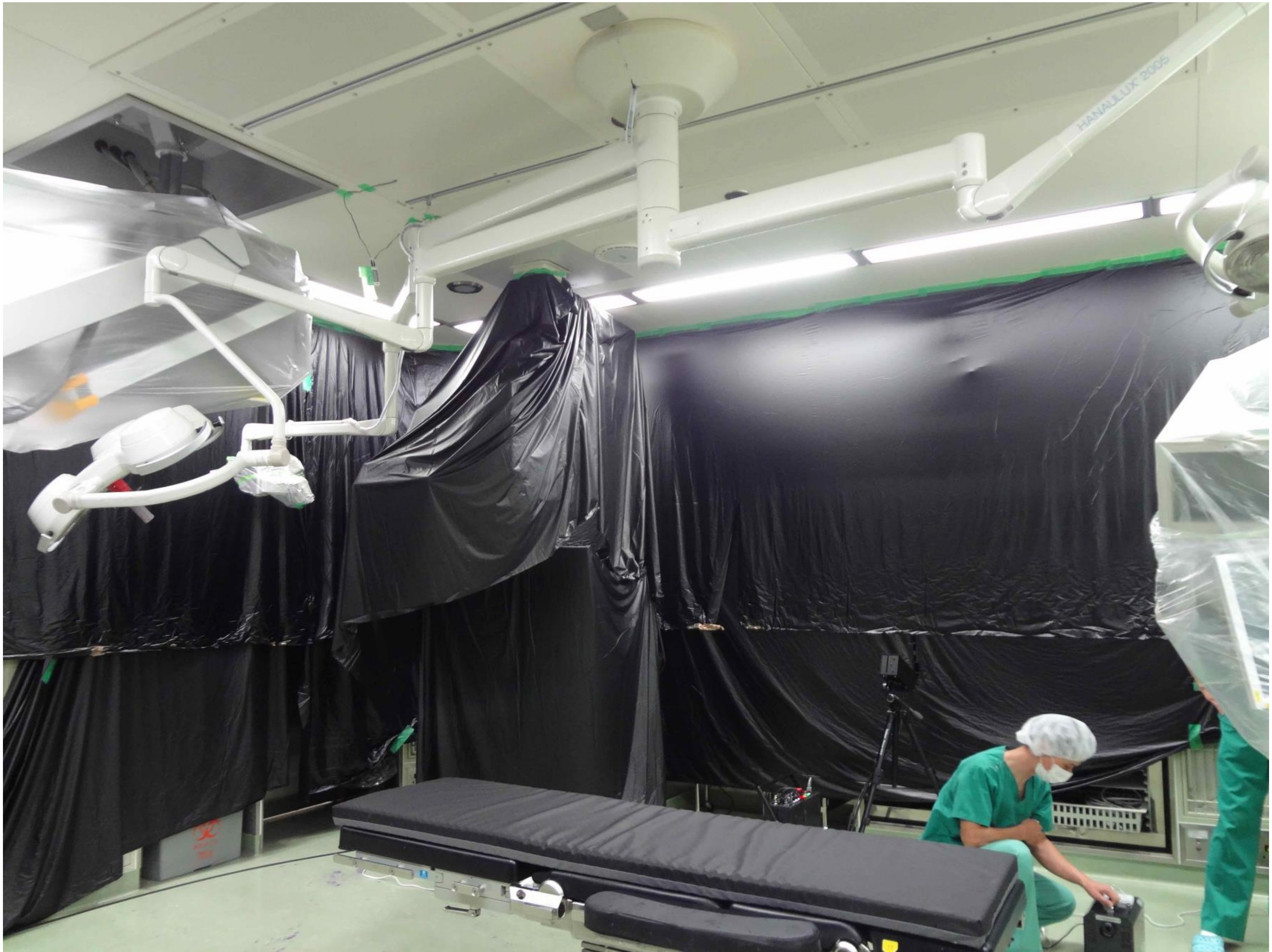

Picture 4s. Preparation for air current visualization in double-axis room with lights in position

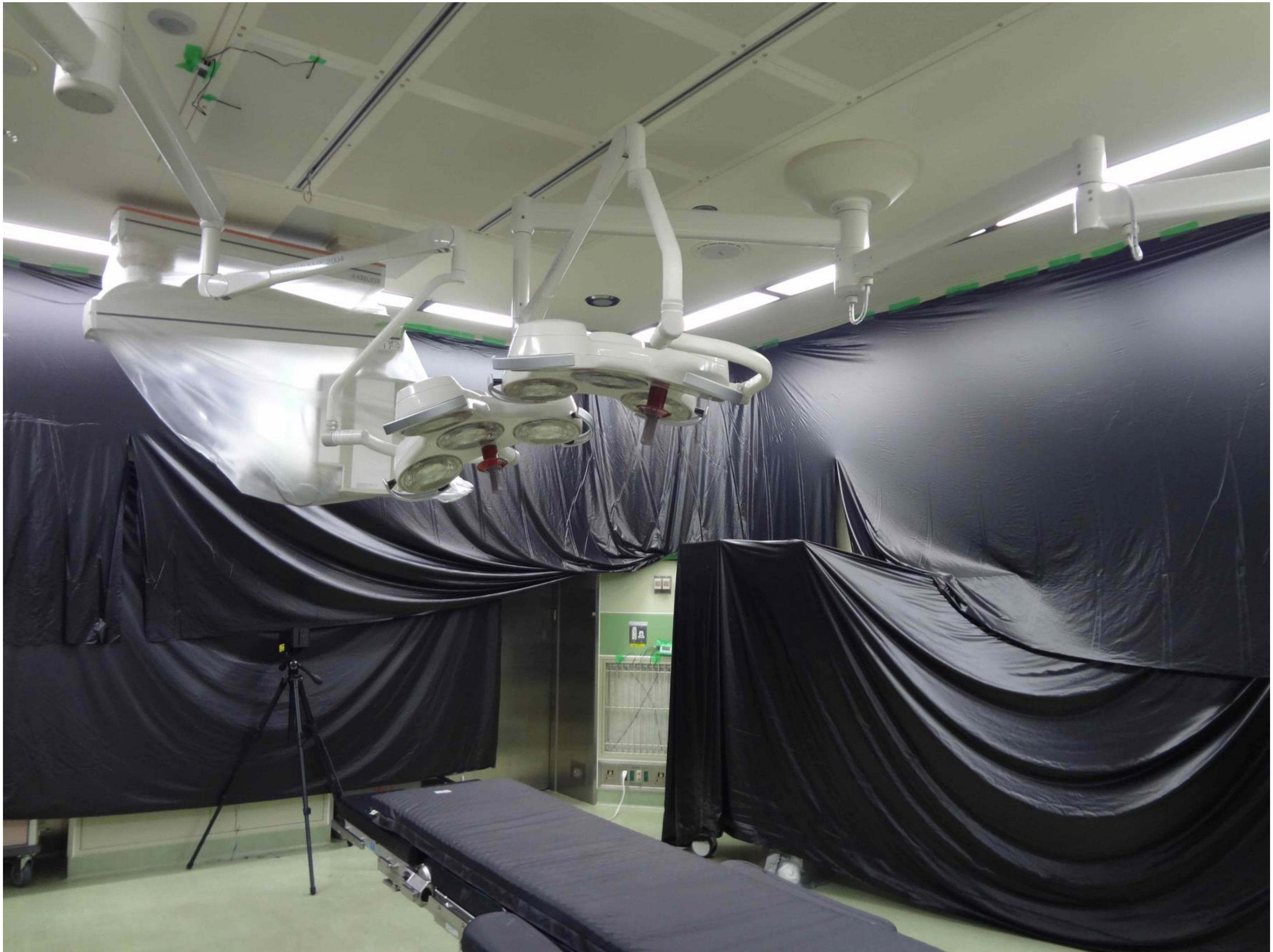

Supplement: Supplementary 3 — Picture 1S: velocity measurement in double-axis room. Picture 2S: cleanliness assessment in single-axis room. Picture 3S: preparation for air current visualization in single-axis room. Picture 4S: preparation for air current visualization in double-axis room with lights in position. [file 4861273.f3.pdf]
